# Evaluation of properties over phylogenetic trees using stochastic logics

**DOI:** 10.1186/s12859-016-1077-7

**Published:** 2016-06-14

**Authors:** José Ignacio Requeno, José Manuel Colom

**Affiliations:** Department of Computer Science and Systems Engineering (DIIS), Universidad de Zaragoza, C/ María de Luna 1, Zaragoza, 50018 Spain

**Keywords:** Phylogenetics, Model checking, Stochastic temporal logic, DNA mutation models, Maximum likelihood

## Abstract

**Background:**

Model checking has been recently introduced as an integrated framework for extracting information of the phylogenetic trees using temporal logics as a querying language, an extension of modal logics that imposes restrictions of a boolean formula along a path of events. The phylogenetic tree is considered a transition system modeling the evolution as a sequence of genomic mutations (we understand mutation as different ways that DNA can be changed), while this kind of logics are suitable for traversing it in a strict and exhaustive way. Given a biological property that we desire to inspect over the phylogeny, the verifier returns true if the specification is satisfied or a counterexample that falsifies it. However, this approach has been only considered over qualitative aspects of the phylogeny.

**Results:**

In this paper, we repair the limitations of the previous framework for including and handling quantitative information such as explicit time or probability. To this end, we apply current probabilistic continuous-time extensions of model checking to phylogenetics. We reinterpret a catalog of qualitative properties in a numerical way, and we also present new properties that couldn’t be analyzed before. For instance, we obtain the likelihood of a tree topology according to a mutation model. As case of study, we analyze several phylogenies in order to obtain the maximum likelihood with the model checking tool PRISM. In addition, we have adapted the software for optimizing the computation of maximum likelihoods.

**Conclusions:**

We have shown that probabilistic model checking is a competitive framework for describing and analyzing quantitative properties over phylogenetic trees. This formalism adds soundness and readability to the definition of models and specifications. Besides, the existence of model checking tools hides the underlying technology, omitting the extension, upgrade, debugging and maintenance of a software tool to the biologists. A set of benchmarks justify the feasibility of our approach.

## Background

A phylogenetic tree is a description of the evolution process which is discovered via molecular sequencing data and morphological data matrices [1]. Computer science tools have upgraded the capabilities of biologists for their construction as well as for extracting and analyzing the implicit biological messages embedded in them [2, 3]. Nowadays, more and more applications rely on the existence of a support phylogenetic tree for the confirmation of biological hypotheses that are valuable for the scientific community. For example, a small but representative portion of these researches combine phylogenetic trees (constructed via the mentioned tools using the information of the genome) with fossils, geographical or phenotypical data in order to find any mismatch, to trace the human migrations [4] and to inspect the distribution of endemic diseases [5]. In this sense, they use a phylogenetic tree for testing biological hypotheses about evolution in a similar way to [6]. Indirectly, the evaluation results and counterexamples help to feedback the phylogeny and increase its quality. However, the wide range of diverse methods and tools used by biologists for studying phylogenetic properties recommended the research of a generic framework for heterogeneous hypotheses testing over trees.

Model checking is a paradigm stemming from computer science based on temporal logics which has been successfully applied in industry for system modeling and verification [7]. The basic principle allowing the use of the model checking framework in the context of phylogeny is the interpretation of the phylogenetic tree as a transition system representing a computational model of the evolution process, i.e., a rooted directed acyclic graph describing the potential behavior of a discrete system whose paths indicate a sequence of intermediate states and the transitions are speciation events. The next step consists of formulating the hypothesis that we desire to investigate over a phylogeny using temporal logics as a formal language. Finally, a model checking tool automatically verifies the satisfaction of the property over the model in an exhaustive way.

Model checking has been recently proposed as a generic unifying framework that allows the phylogeneticist to focus on tree structures, biological properties and symbolic manipulation of phylogenies described using temporal logic, instead of on implementation issues concerned with verification algorithms [8, 9]. The model checking framework helps us to uncouple software tools from the formal definition of models and the specification of properties. Besides, it hides the underlying implementation technology to final users, enabling transparent software upgrades of the model checking tool and removing debugging and programming language concerns through different platforms [10]. Standard branching-time temporal logics such as *Computational Tree Logic* (CTL [11]) already allow expressing biological properties referred to the structure of the tree or the arrangement of DNA sequences along the paths [8].

In this paper, we repair the limitations of the previous model checking approach using standard temporal logics. Some of the phylogenetic hypotheses require a more powerful language for being described, i.e., it is necessary to extended the models and specification logics with explicit time and probabilities. Evolution is a continuous-time and stochastic process where the branch length of a phylogeny represents time, and the distribution of biological features is non-deterministic along the whole tree. One of the potential applications of probabilistic model checking is the study of the dispersion of lactose intolerance among populations and the temporal point when it appeared in the tree [12]. The objective is to tolerate more flexibility and expressiveness in the specification of properties.

Explicit time and probabilities are not directly supported by qualitative logics, but they can be included in the specifications of a probabilistic transition system using discrete (PCTL [13]) or continuous-time logics (CSL^TA^ [14]). Much more logics have been proposed according to different metrics and time models [15] (e.g., number of clocks in the system or local vs global clocks), although we will focus on CSL^TA^ here. The interpretation of this kind of specifications requires the modification of the traditional transition system and the algorithms that traverse them. The models must be enlarged in order to support these new informations.

More in detail, we present and classify a more complete description of phylogenetic properties that are intrinsically stochastic by nature. Our aim is to show that an extended model checking framework is suitable for solving temporal and probabilistic problems in the domain of phylogenetics. Current probabilistic model checking tools are generic and powerful enough for our proposal. Other tools such as Phycas [16], RevBayes [17] or Beast2 [18] are focused on solving a set of specific problems while our approach can answer a wider range of questions.

The advantages of applying the probabilistic continuous-time extensions of model checking techniques for evaluating phylogenetic specifications are twofold: a) it is possible to verify if a property is true or false according to some temporal requirements and the probabilistic behavior of the model and b) it is also possible to obtain the minimum probability that makes the specification true within an interval of time. In order to show the feasibility of our approach, we apply our framework to a specific problem: the probability of obtaining a phylogeny with a certain topology. Specially, we focus on the computation of maximum likelihood estimations (MLE) for a phylogenetic tree using models of DNA evolution, that simulate the changes in the genome through the time.

The paper is arranged as follows. The next Section “Methodology” introduces the main notions of evolution and explains how it is modeled under the model checking framework. Besides, the logical specification of nontrivial phylogenetic properties is described. The logic presented in this section is used for evaluating stochastic properties and the likelihood of phylogenetic trees in the case of study. Section “Results and discussion” introduces the methods for managing continuous-time and probabilities in model checking and current probabilistic verifiers. After that, the case of study with maximum likelihood of a phylogenetic tree, and the experimentation details are presented. The key steps for implementing phylogenetic trees and biological properties within the scope of the PRISM tool is presented. Finally, Section “Conclusions” gathers the conclusions drawn from this research and details the future work.

## Methodology

### Analyzing models of evolution with probabilistic model checking

The process of model checking is divided in three phases. The first part consists of a correct modeling of the system. Next, a set of properties are written using a specification logic. Finally, a model checking tool takes the description of the model and the hypotheses, and analyze them in an automatic and exhaustive way. The quality of the analysis is influenced by the identification of the main characteristics of the system during modeling. To this end, the next paragraphs present the principal keystones that we try to capture of the evolution model and how they are translated into a computational model (i.e., transition system) suitable for the study.

#### Evolution as a transition system

Evolution is an infinite-time, not-ending process that keeps introducing new traits and species forever. It can be modeled at different levels of abstraction. Depending on the aspects we focus on, evolution can be simulated by:

- **Topological models**. Birth-death Markovian models describe the macroevolution process of speciation [19, 20]. They are usually used for the generation of random trees fulfilling the topologies and structural characteristics that are supposed to appear in correct phylogenies. They provide a skeleton (i.e., a phylogenetic structure) regardless of the internal details of the tree states. These trees would be later enriched by complementary information (e.g., DNA). These trees, or those constructed by inference or maximum parsimony methods [21], are used as initial seed in subsequent problems, for example, for the tree exploration phase during the evaluation of the maximum likelihood estimations [22, 23].
- **Sequence models**. On the other hand, the genome constitutes nowadays one of the most important traits of a species for its study. Models that describe the modification of protein or DNA sequences through time provide finer details about the genetic changes operating behind the macroevolution models. These models nuance the process from which a sequence of characters switches into another one. In short, the sequence models capture how the genomic information is placed in each state of a tree inferred from a speciation model.

**Fig. 1:**
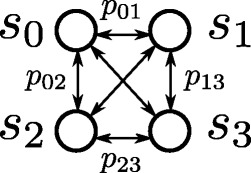
Model of DNA mutation

Often, the phylogeneticists identify the nucleic changes as the main source of the macroevolution process and then associate the notion of evolution history to the concept of mutation trace. Hence, DNA mutation models are commonly referred to as the true tree generators or *phylogenetic models* in the literature: the phylogenies arise as the result of a spanning simulation of these mutation models embedding the complex process of speciation. At this point, it is not surprising that biologists try to match the tree of life that they inferred from an input alignment and reconstruction method with the execution of an instance of a DNA mutation model that could generate it. Figure 1 illustrates a simple mutation model where state *s*_*i*_ represents a nucleotide at a certain position of the sequence and it has a probability *p*_*ij*_ of changing to state (nucleotide) *s*_*j*_. Some probabilities are omitted for readability.

In the context of molecular evolution, the phylogenetic tree would indicate a *reachability graph* or *execution trace* of the system, that is, a snapshot capturing the order of occurrence of the mutations along the history of the speciation process since the beginning of time until present day. Therefore, a phylogenetic tree will reflect how the mutation model should act, showing the consecutive changes in a long-term run of the model. In other words, the phylogeny represents the *behavior* of the system, understood as a computational process modeling the hypothetical flow of the DNA during the time.

**Fig. 2:**
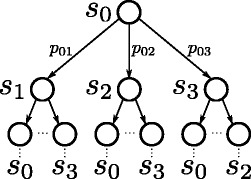
Unfolding of a DNA mutation model in a phylogenetic tree

Figure 2 shows a *phylogenetic tree* generated by the unfolding of a mutation model for a single position of the DNA string. A complete strand is simulated by the concatenation of *n* (theoretically) independent models of evolution, one per position. A phylogenetic tree for the whole genome has a n-dimensional array of characters (bases) for each state and a n-dimensional array of probabilities for each transition. The probability of moving from a state *s*_*j*_ to a state *s*_*k*_ is calculated according to the vector associated to the transition. This value is equal to $\Pi _{i=1}^{n}p_{jk}(i)$ with *p*_*jk*_(*i*) denoting the probability of changing a nucleotide *s*_*j*_(*i*) by a *s*_*k*_(*i*) at the i’th position of the array. Transitions with null probability are removed from the tree.

**Fig. 3:**
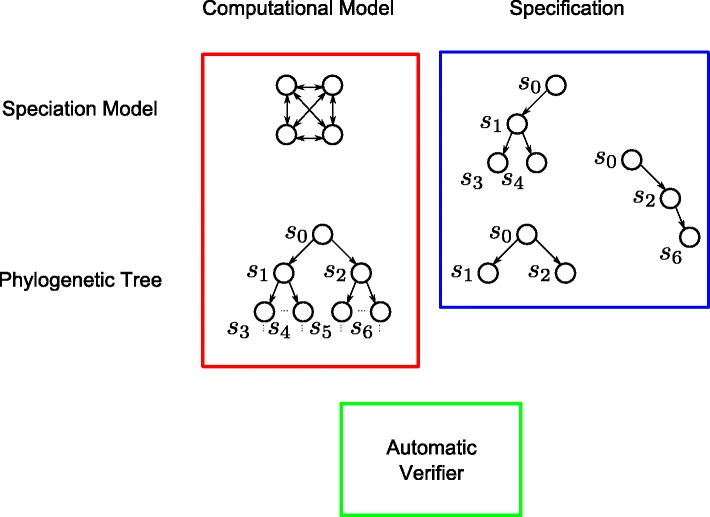
Probabilistic model checking framework in phylogenetics

In a computer science context, both speciation models and phylogenies are two faces of the same coin and they can be used as *computational models* expressing the behavior of evolution (Fig. 3). Later on, a set of properties are studied over the model using an adequate description language such as temporal logics. A temporal logic allows, using a certain symbolism and rules, reasoning about propositions qualified in terms of abstract time (i.e., time in the sense of partial ordering of the events). This kind of logics focus on the information stored in the states and the relationship among the nodes. For example, it is possible to declare questions such as “eventually in the future, a mutation of *σ* to *α* happens in a branch of the tree”, or “generally, position *i* is conserved in this haplogroup or subtree”. A verifier takes the propositions expressed in temporal logic and a model described as a transition system, and tells if they are true or not for this particular context. It explores the reachability graph in an automatic and exhaustive way.

#### Markov chain as a data structure

In probabilistic model checking, the computational models are probabilistic transition systems (PTSs). By default, topological and sequence models are Markov chains whose structure and semantics matches directly with PTSs. For example, the simpler models of evolution leading to the introduction of mutations in the genome are a four-state Markov chain with different probabilities for the transitions among nucleotides [24]. Commonly, they are parameterized templates indicating the ratios and relations between bases. Several versions of DNA mutation models have been proposed in the literature. By now, there are highly optimized models for specific purposes including the special biochemical features of the nucleotides such as physical-chemical stability of the strands.

Markov chains accept either discrete (integer) or continuous (real) time in the modeling, thought continuous-time is more suitable for capturing finer temporal details of the evolution. Instead of describing explicitly the probability of change from one state to another, the transitions are labeled with rates delimiting the time spent in that branch.

##### **Definition1** (Continuous-time Markov Chain).

A continuous-time Markov chain (CTMC) is a finite transition system represented by a tuple *M*=(*S,S*_0_,**R**,*L*), where:

- *S* is a finite set of states,
- *S*_0_⊆*S* is the set of initial states,
- $\mathbf {R} \subseteq S \times S \rightarrow \mathbb {R}_{\geq 0}$ is the transition rate matrix between states, i.e., for every pair of states *s,s*^′^∈*S*, a transition occurs only if **R**(*s,s*^′^)>0, and the probability of this transition being triggered in *t* time units equals $1-e^{-\mathbf {R}(s,s')\cdot t}\phantom {\dot {i}\!}$, and
- *L*:*S*→2^*AP*^ is the labeling function that associates each state with the subset of atomic propositions (*AP*) that are true of it.

**Fig. 4:**
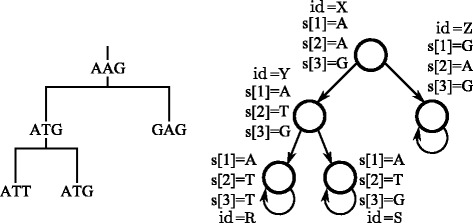
Translation from a phylogenetic tree to a probabilistic transition system

An atomic proposition is a declarative statement telling the properties of a state of the model. It may cover several aspects of the evolution (location, disease or phenotype of species and individuals), thought the simpler one is the nucleic value. For instance, the set of atomic propositions assigned to the states in Fig. 4 corresponds to the nucleic values of the genome in those points.

As there exist multiple pair candidates with **R**(*s,s*^′^)>0 outgoing from state *s*, a race condition appears and the selection of the next state determines the history of events. A higher value of **R**(*s,s*^′^) prioritizes the transition between *s* and *s*^′^. If all the successors share the same ratio, the transitions are equally probable. The elapsed time before triggering affects the probability of a transition.

The computation of probabilities depends on the paths starting in the initial state. Given an infinite path of successive states *π*=*s*_0_*s*_1_*s*_2_…, *π*[*i*]=*s*_*i*_, while *π*(*t*) returns the state *s*_*i*_ of the path in which the system is found after $t \in \mathbb {R}_{\geq 0}$ time units since the initial state *s*_0_ (i.e., *π*[0]=*π*(0)=*s*_0_). For any set of infinite paths *Π* starting in the initial state *s*_0_, the subset *Π*(*π*_*n*_) selects the paths *π*∈*Π* whose prefix equals to the finite sequence *π*_*n*_=*s*_0_*s*_1_*s*_2_…*s*_*n*_ of length *n*+1 states.

The likelihood of reaching a state *s*_*n*_ is determined by the probability measure *Pr*(*Π*(*π*_*n*_))=*Pr*(*Π*(*s*_0_*s*_1_*s*_2_…*s*_*n*_)) over all the paths sharing the prefix *π*_*n*_ with *π*_*n*_(0)=*s*_0_. For the trivial case over *Π*(*s*_0_), *Pr*(*Π*(*s*_0_))=1. In general for *Π*(*π*_*n*_), *Pr*(*Π*(*s*_0_*s*_1_*s*_2_…*s*_*n*_))=**P**_*t*_(*s,s*_*n*_). Starting in *s*, the probability of arriving to a state *s*^′^ at a particular time instant *t* is represented by **P**_*t*_(*s,s*^′^)=*Pr*{*π*∈*Π* | *π*(*t*)=*s*^′^}, with **P**_*t*_ an exponential matrix indicating the transient probabilities for time *t* and **Q** the infinitesimal generator matrix of *M*:

$$\mathbf{P}_{t} = e^{\mathbf{Q} \cdot t} = \Sigma_{i=0}^{\infty} \frac{(\mathbf{Q} \cdot t)^{i}}{i!} $$

The infinitesimal generator matrix **Q** is used for the calculation of transient states and it is defined as:

$$\mathbf{Q}(s,s') = \left\{ \begin{array}{l l} \mathbf{R}(s,s') & \quad \text{if }s \neq s'\\ - \Sigma_{s^{\prime\prime} \neq s} \mathbf{R}(s,s^{\prime\prime}) & \quad \text{otherwise} \end{array} \right. $$

An alternative and more stable standard technique for calculating the probability matrix **P**_*t*_(*s,s*^′^) is presented in [25].

Assuming that phylogenetic trees are the state spaces produced by the executions of evolution models, they are also accepted as PTSs and Markov chains. Each transition connecting two nodes of the tree has the same information, probability and temporal requirements than the transition connecting an equivalent pair of states of the evolution model. Self-loops with unitary probability are printed in final states of the tree to produce infinite traces of present-day taxa because of uncertainty of future traits and species (Fig. 4). This trick is necessary for evaluating temporal properties and imposing conditions for the mathematical resolution of probabilities. The theoretical concepts, logics and tools will be detailed in the following sections.

### Specification of phylogenetic properties over phylogenies

During the last years, new formalisms and tools have started to accept properties expressed in probabilistic temporal logics for capturing and analyzing the underlying randomness of stochastic systems. Probabilistic model checking combines the expressiveness for representing paths of a computational model using temporal logics with the calculation of likelihoods associated to the route. Our objective is to enrich the original applications presented in [8] with probabilities and explicit time in order to obtain a more flexible framework capable of solving complex problems.

#### Continuous-time probabilistic logic as a specification language

Once the data structure and semantic of the model are defined, the next step involves the presentation of the syntax and semantics for the stochastic temporal logic. The logic proposed here for working with CTMCs is CSL^TA^ [14].

##### **Definition2** (Continuous-time Stochastic Logic).

A temporal logic formula *ϕ*and a path formula *Φ*are defined by the following minimal grammar, where *p*∈*AP*, *λ*∈[0,1] is a real number denoting a probability, and ∼ in {<,≤,=,>,≥} is a comparison operator:

1 $$\begin{array}{@{}rcl@{}} \phi & ::= & true \:|\: p \:|\: \neg \phi \:|\: \phi \vee \phi \:|\: \mathbb{P}_{\sim \lambda} \left[ \Phi \right] \\ \Phi & ::= & \mathbf{X} \phi \:|\: \left[\phi \mathbf{U}_{I} \phi \right]  \end{array} $$

The formulas are checked against a structure *M* considering all paths *π* emerging from a state *s*_0_. Notice that $M, s_{0} \vDash \phi $ means that *s*_0_ satisfies *ϕ*. The semantics of well-formed formulas is as follows (let *π*=*s*_0_*s*_1_*s*_2_…):

- $M, s_{0} \vDash p \Leftrightarrow p \in L \left (s_{0} \right)$,
- $M, s_{0} \vDash \neg \phi \Leftrightarrow M, s_{0} \nvDash \phi $,
- $M, s_{0} \vDash \phi \vee \psi \Leftrightarrow M, s_{0} \vDash \phi $ or $M, s_{0} \vDash \psi $,
- $M, s_{0} \vDash \mathbb {P}_{\sim \lambda } \left [ \Phi \right ] \Leftrightarrow Prob(M,s_{0},\Phi) \sim \lambda $,

The calculation of the probability *Prob*(*M,s*_0_,*Φ*) requires the identification of the set of infinite paths *π* satisfying the path formula $M, \pi \vDash \Phi $:

- $ M,\pi \vDash \mathbf {X} \phi \Leftrightarrow M,\pi [1] \vDash \phi $
- $M,\pi \vDash \left [ \phi \mathbf {U}_{I} \psi \right ] \Leftrightarrow $ for some *t*∈*I*, $\exists \pi : M, \pi (t) \vDash \psi $, and $M, \pi (t') \vDash \phi $ for all 0≤*t*^′^<*t* with *I* an interval of reals.

The set $\{\pi \in \Pi \:|\: M, \pi \vDash \Phi \}$ is obtained by the union of finitely many pairwise disjoint subsets *Π*(*π*_*n*_) by ([26] Definition 3), each one characterized by the finite prefix *π*_*n*_ of all infinite sequences of the set satisfying *Φ*. Therefore, the probability function *Prob*(*M,s*_0_,*Φ*) is computed as the summation of probabilities in all possible prefixes *π*_*n*_ by ([26] Theorem 1). That is, $Prob(M, s_{0},\Phi) = \Sigma _{\pi _{n}} Pr(\Pi (\pi _{n}))\phantom {\dot {i}\!}$ where *Pr* is a specialized function for obtaining the probability of the path defined by *π*_*n*_. The calculation of the probability will be detailed in a further section.

In qualitative branching-time logics such as CTL, every path formula imposes a reachability relation or patterns among all the states that satisfy some propositional requirements. That is, they define sequences of events that must be found in the model. CTL reinterprets the quantifiers of first-order logic as *path quantifiers*, expressing the fulfillment of a property throughout all computation paths (**A**), or at least exists one computation path (**E**). These two must be immediately qualified by one of five *temporal operators*, of which three express the satisfaction of a property eventually in a future state (**F**), generally at all states (**G**), or in the next state (**X**); and two are conditional constructs in which a precedent is verified until a consequent comes into force (**U**), or until and including the moment when it does, if it does (**R**). A complete grammar and semantics of CTL formulas can be defined from a minimal subset of logical operators [27].

**Fig. 5:**
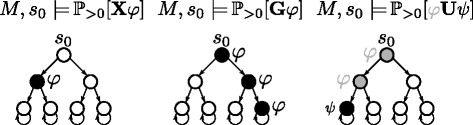
Evaluation of probabilistic temporal logic operators

In probabilistic temporal logics, path quantifiers are substituted by probabilistic operators. The satisfaction of a propositional over all (**A**) paths is equivalent to maximize the expected probability ($\mathbb {P}_{\geq 1}$). The existence (**E**) of at least one path imposes the probability to be greater than zero ($\mathbb {P}_{> 0}$). Figure 5 describes graphically the basic semantic.

CSL^TA^ supports timed transitions in the **U** operator. Timed variants of the modal operators **F** and **G** are obtained via **U** as **F**_*I*_*ϕ*=*true***U**_*I*_*ϕ* and **G**_*I*_*ϕ*=¬**F**_*I*_¬*ϕ*. Instead of writing probability and intervals explicitly, they can be abbreviated with inequalities. For example, $\mathbb {P}_{\leq 0.5}\left [\Phi \right ]$ denotes $\mathbb {P}_{[0,0.5]}\left [\Phi \right ]$. This temporal extension constrains the length of the computational path specified in the logical equation. The operator **X** implicitly takes the real time elapsed for a consecutive branch of the current state. By now, phylogenetic properties querying about the structure of the tree or mutation traces can be expressed in CSL^TA^.

#### Sequence properties

In a non-probabilistic temporal logic, it is already possible to query a wide range of questions about the arrangement of the nucleic information in the states of the tree [8]. However, what we look for here is the addition of probabilistic information to the specifications. For instance, a preliminary probabilistic study focused on lactose intolerance within humans [12]: it identifies the haplogroups having a higher concentration of SNP’s associated to this disease in the leaves of the phylogenetic tree.

In a similar way to evolution models, phylogenetic properties can be classified as *sequence properties* or *topological properties* depending on the information they extract from the phylogeny. In general, sequence properties are based on the content of the states, i.e., those propositions that are evaluated within a node and without need of temporal operators. Usually, they are composed of simple patterns whose application scope is restricted to the surrounding nodes or the entire phylogeny. Such types of state formulas are called *patterns(p,t)*, being *p* the propositions that must be true and *t* an optional temporal parameter (*t*=*∞* when omitted). They provide a powerful descriptive formalism for formulating general restrictions without the limitations of ad hoc approaches. Often, these properties may be used not necessarily to forbid patterns, but as queries and alerts to signal unusual, possibly anomalous behavior, and mark it for further study.

**Patterns in the genome**. A first group of patterns represents global correctness constraints that are supposed to hold across the whole phylogeny. They can be categorized as follows:

- *Conservation* is modeled as a restriction on the symbols that can occur at a given position in a sequence. Commonly, the pattern is codified according to a set of (im)permissible symbols. It is possible to define general families of compatible elements, not bounded to specific positions, and restrict their usage to exactly one of these positions when needed.
- *Covariation* imposes a relation of dependence between two (or more) positions in a sequence due to some physical-chemical constraints or the internal structure of the genome. It represents the set of symbols that may appear in the second place in association to each symbol in the first place.

A global (**G**) pattern thus defined is easily verified by extending it over the computation tree.

$$global \left(p, t \right) \equiv \mathbb{P}_{\geq 1} \left[ \mathbf{G}_{\leq t} \left(p \right) \right] $$

The variation of the probabilistic parameter determines the scope (i.e., width) of the pattern over the complete state space. Lineage-specific haplogroup verification represents a further step forward, where patterns would be used to define *conditional explorations* of the sets of states of interest of the phylogeny.

**Deleterious mutations**. Exceptions to the aforementioned properties may in fact indicate suspicious or potentially deleterious mutations, which are of great interest in applied phylogenetic studies [28]. Furthermore, known or suspected mutations of this kind can be explicitly modeled as patterns and their positioning in a phylogeny assessed. In particular, those affecting important metabolic functions are expected to prevent or hinder the reproduction of the organism, and consequently should be confined in or near terminal leaves.

Observed and feasible deleterious mutations may be permitted subject to certain restrictions. Specifically, it may be demanded that, if a hazardous pattern *p* appears, it has no offspring, i.e., it is a leaf in the phylogeny; or, to provide some flexibility, it may be allowed that all descendants, if any, are reached in at most time *t* ($\mathbb {P}_{\geq 1} \left [ \mathbf {F}_{\leq t} \right ] $).

$$terminal \left(p \right) \equiv \mathbb{P}_{\geq 1} \left[ \mathbf{G} \left(p \implies leaf \right) \right] $$

$$terminal \left(p, t \right) \equiv \mathbb{P}_{\geq 1} \left[ \mathbf{G} \left(p \implies \mathbb{P}_{\geq 1} \left[ \mathbf{F}_{\leq t} \left(leaf \right) \right] \right) \right] $$

In this case, leaves (self-loops in the phylogenetic structure) must be detected without reference to any particular sequence. This is easily achieved by performing an equality comparison between the valuations of *AP* of the target state and all its successors.

$$leaf \equiv {\textstyle \bigwedge_{prop \in AP}} p \Leftrightarrow \mathbb{P}_{\geq 1} \left[ \mathbf{X} \left(prop \right) \right] $$

**Point mutations**. Point mutations, reversions and parallel or convergent evolution of sequences are properties that are arranged over local paths of the tree. Thus, they need access to the topological structure of the tree in some sense. It is reasonable to start considering a relatively simple properties based on a tree topology and an associated sequence alignment, exemplified by a point mutation. For legibility and compactness in the following example, we will refer to the atomic propositions *s*[*i*]=*σ* (*s*[*i*]≠*σ*) of a state as *σ*_*i*_ ($\overline {\sigma }_{i}$). Suppose for now that the alignment comprises a number of characters indexed 1 through *l*, sequences *s* are words of length *n* over an alphabet *Σ*, and *s*[*i*]=*σ* (*s*[*i*]≠*σ*) means that *σ*∈*Σ* appears (not) in position *i* in a state sequence (recall Definition 1 and Fig. 4). A point mutation is represented by the equation:

$$\mathbb{P}_{\geq \lambda} \left[ \sigma_{i} \wedge \left(\mathbf{X} \, \alpha_{i} \right) \right] $$

which tells whether *σ* is replaced by *α* at position *i* in the next state with a probability greater than *λ*.

**Back mutations**. It is possible to determine whether a given tree is free of back mutations or reversions, which we abbreviate *BM*. Given an internal node, there is a back mutation in that subtree involving a position *j* of the alignment if at some point in some descending path from the node we find a different symbol ($\overline {\sigma }_{\mathit {j}}$) than that found in the root of the subtree (*σ*_*j*_). Then, at some point in the subtree hanging from that intermediate the symbol from the root reoccurs.

The formula must model this condition by nesting **F** operators: a node satisfies a property which a) eventually some other descendant does not satisfy, but b) it is fulfilled once again at some point in the future. The check is repeated for every symbol that may occur in the node.

$$\mathit{hasBM} \left(\mathit{col} \right) \equiv \bigvee_{\sigma \in \Sigma} \sigma_{\mathit{col}} \wedge \mathbb{P}_{> 0} \left[ \mathbf{F} \left(\overline{\sigma}_{\mathit{col}} \wedge \mathbb{P}_{> 0} \left[ \mathbf{F} \left(\sigma_{\mathit{col}} \right) \right] \right) \right] $$

Finally, the global formula *detectBM* iterates the check over the positions of the alignment and extends it to all tree nodes.

$$\mathit{detectBM} \equiv {\textstyle \bigwedge_{j = 1}^{l}} \mathbb{P}_{\geq 1} \left[ \mathbf{G} \left(\neg \mathit{hasBM} \left(j \right) \right) \right] $$

In these two formulas we present a non-trivial modeling example of a cladistic property with a heavy use of sequence data. The goal is to detect if the tree is free of back mutations (equivalently, it detects those points in the tree where back mutations occur due to counterexamples, if any). In the case of finite domains, such as the set of DNA sequences, the evaluation of logical quantifiers ∀ and ∃ can be substituted by multiple instances of boolean formulas connected by the $\bigwedge $ and $\bigvee $ operators. In sum, we have formalized the concept of back mutation in probabilistic temporal logic. The addition of intervals refine the length of the paths in the property.

#### Topological properties

Identifying if a phylogenetic tree reflects the correct structure of the evolution is one of the most important topological questions. Given a phylogenetic tree inferred from an alignment, it should be compared to the speciation model that we use as reference, for instance, a DNA mutation model. The computation of maximum likelihoods quantifies the fitness or probability that the topology obtained by the alignment follows the same trace imposed by a mutation model [29].

Due to the uncertainty of the *true* evolution process generating the tree of life, the estimation of maximum likelihoods works as a metric for comparing the topology of two or more trees built over the same data set (the higher value for this score, the better). The selection of other inferred phylogenies, alignments or alternative DNA models modifies the probabilities of the arrangements and returns different scores [30].

The computation of the likelihood score is carried out by the following formulas, which will be later composed as logical equations. At position *l* of a sequence with length *n*, the probability of changing from nucleotide *i* in state *X* to nucleotide *j* in state *Y* is denoted by the probability function *P*_*ij*_(*d*_*XY*_). The variable *d*_*XY*_ indicates the branch (temporal) length between the states *X* and *Y*, which is a positive value for each pair of connected nodes. Equation 2 corresponds to the overall likelihood for the tree in Fig. 4. The probability function *P*_*ij*_(·) depends on the selected mutation model [29, 31, 32]. This probability function is determined by the model checking tool by analyzing the transitions of the mutation model that it uses as computational model. The expression *Z*(*l*) denotes the base of the DNA in taxon *Z* at position *l*. The constant *f*_*i*_ is the theoretical frequency of the base *i* in the alignment, which is dependent of the mutation model.

2 $$\begin{array}{@{}rcl@{}}  L^{l} = \Sigma_{i=\{A,C,G,T\}}\Sigma_{j=\{A,C,G,T\}} & f_{i}P_{ij}(d_{XY})P_{iZ(l)}(d_{XZ}) & \\  & P_{jR(l)}(d_{YR})P_{jS(l)}(d_{YS}) & \end{array} $$

Equation 2 can be factorized as the combination of the likelihood of the left and right subtrees in order to avoid the recalculation of the same subformula multiple times:

3 $$\begin{array}{@{}rcl@{}}  {L^{l}_{X}} & = & \Sigma_{i=\{A, C, G, T\}} L^{l}_{X,i} \end{array} $$

4 $$\begin{array}{@{}rcl@{}} L^{l}_{X,i} & = & \Sigma_{j}P_{ij}(d_{XY})L^{l}_{Y,j} \cdot \Sigma_{k}P_{ik}(d_{XZ})L^{l}_{Z,k} \end{array} $$

Normally, the DNA of present-time taxa in the leaves of the tree has maximum likelihood (i.e., $L^{1}_{Y,A}=1$). In opposition, the nucleic bases of the internal nodes are unknown and they shall be inferred with the mentioned formulas. Due to the uncertainty in the genome of the ancestral states, the estimation of the likelihood in *X* at position *l* implies the summation of $L^{l}_{X,i}$ for all the possible nucleotides. The evaluation of the likelihood equation at the root node returns the score *L*_*Root*_ for the whole tree. The likelihood value of a complete genomic sequence corresponds to the product of the likelihood values of every position since we assume that sites evolve independently of each other. Logarithms over *L*_*Root*_ are regularly used because of the small likelihood values.

5 $$\begin{array}{@{}rcl@{}}  L^{l}_{Root} & = & \Sigma_{i=\{A, C, G, T\}} f_{i} L^{l}_{Root,i} \end{array} $$

6 $$\begin{array}{@{}rcl@{}} L_{Root} & = & \Pi_{l=1}^{l=n} L^{l}_{Root} \end{array} $$

Nevertheless, the search for the tree with maximum likelihood requires the evaluation of a considerable number of equations over a great tree space, which converts the searching process using scores in an NP-hard problem [1]. Despite the storage of partial solutions ($L^{l}_{X,i}$), more heuristics must be introduced in order to gain feasible solutions.

Extra simplifications consist of a) the propagation of the local maximum $L^{l}_{X,i}$ instead of the four internal values $L^{l}_{X,i}$ and b) the assignation of a preliminary character to the internal ancestors of the phylogenetic tree (for instance, using a maximum parsimony method when there is no ambiguity in the selection of the nucleotide). The main drawback of these approaches is the extraction of a local maximum instead of the real solution. The selection of the initial seed and the intensive sampling method of the tree space determines the quality of the approximation to the real likelihood value for the phylogeny [33].

In Section “Case of study: maximum likelihood of a phylogenetic tree” we show how a model checking tool can test and compute the reliability of a tree according to the defined mutation model. The mathematical equations of MLE for evaluating the phylogeny are rewritten using probabilistic temporal logics. Later, they are executed over a CTMC chain corresponding to a DNA mutation model. Finally, the model checking procedure queries whether the probability of appearance of a particular mutation in the tree is over or not a predefined threshold. The evaluation of the CSL^TA^ formulas needs the procedure for computing probabilities introduced in the next section.

## Results and discussion

### Algorithm and tools for model checking

The evaluation of biological hypotheses qualified with probability and time requires the extension of the traditional model checking algorithms [27]. The first point of this section is devoted to the introduction of functions and numerical equations for solving the quantitative part of the formulas, which is the main difference with respect to previous model checking algorithms. The second point focus on the tools implementing this extension.

#### Algorithm for CSL^TA^

Given a PTS and a CSL^TA^ formula *ϕ*, the model checking problem consists of identifying the set of states where *ϕ* is valid. The basic procedure implies a recursive computation of the state set *Sat*(*ϕ*) for all the states satisfying *ϕ*. The model checking algorithms for solving CSL^TA^ formulas are mainly identical to those of classic model checking [27] except for the resolution of probabilities in $\mathbb {P}_{\sim \lambda }[\Phi ]$. In short, the recursive algorithm of probabilistic model checking incorporates the new sentence:

$$Sat(\mathbb{P}_{\sim \lambda}\left[\Phi\right])=\{ s \in S \:|\: Prob(M, s, \Phi) \sim \lambda \} $$

The computation of the *Prob* function sometimes requires a discretized version of the CTMC. The embedded discrete-time Markov chain (DTMC) is the same tuple *M* (Definition 1) but replacing the transition rate matrix **R** by a transition probability matrix **P** whose values are:

$$\mathbf{P}_{emb(M)}(s,s') = \left\{ \begin{array}{l l} \frac{\mathbf{R}(s,s')}{E(s)} & \quad \text{if }E(s) \neq 0\\ 1 & \quad \text{if }E(s) = 0\,\, \text{and\,\,} s=s'\\ 0 & \quad \text{otherwise} \end{array} \right. $$

*E*(*s*) is known as the *exit rate* of state *s*. It is defined as the summation of every output transition rate **R**(*s,s*^′^):

$$E(s)= \Sigma_{s' \in S} \mathbf{R}(s,s') $$

The value *E*(*s*)=0 means that *s* is an *absorbing state* or *siphon*. The path probability for each path operator is now calculated as follows.

##### $\mathbb {P}_{\sim \lambda }[ \mathbf {X} \phi ]$ formula.

In CSL^TA^, the next operator has no sense as in continuous-time there is not an unique next real number. This operator is included for compatibility. It detects if any direct transition between a state *s* and a successor *s*^′^ has probability **P**_*emb*(*M*)_(*s,s*^′^)∼*λ* in the embedded DTMC.

##### $\mathbb {P}_{\sim \lambda }[ \psi \mathbf {U}_{I} \phi ]$ formula.

The computation of the probability for the until operator depends on the value of the interval *I*. Generally, the interval *I* is classified as:

- *I*=[0,*t*] with $t\in \mathbb {R}_{\geq 0}$;
- *I*=[*t,t*^′^] with $t, t'\in \mathbb {R}_{\geq 0}$ and *t*≤*t*^′^;
- *I*=[*t*,*∞*] with $t\in \mathbb {R}_{\geq 0}$.

For the case of *I* = [0,*t*], the probability *Prob*(*M,s*,*ψ***U**_[0,*t*]_*ϕ*) is equal to: **(i)** 1, if *s*∈*Sat*(*ϕ*)**(ii)** 0, if *s*∈*Sat*(¬*ψ*∧¬*ϕ*) or *t*=0**(iii)**$\Sigma _{\substack {s \in Sat(\psi) \\ s' \in Sat(\phi)}} \mathbf {P}_{t}(s,s')\phantom {\dot {i}\!}$, otherwise

When *I*= [0,*∞*], then *Prob*(*M,s*,*ψ***U**_[0,*∞*]_*ϕ*)=*Prob*(*emb*(*M*),*s*,*ψ***U***ϕ*). It is calculated indefinitely as follows until a stop condition is reached (i-ii):

$$\begin{aligned} {}Prob(emb&(M), s, \psi \mathbf{U} \phi)\\ =& \Sigma_{\underset{s' \in Sat(\psi \vee \phi)} {s \in Sat(\psi)}}\mathbf{P}_{emb(M)}(s,s') \cdot Prob(emb(M),s',\psi \mathbf{U} \phi) \end{aligned} $$

The embedded DTMC must be free of loops (infinite paths) of intermediate states so as to have a finite and solvable numerical equation system.

When *I*= [*t,t*^′^], the until operator must consider a) the time *t* spent in states satisfying *ψ* plus b) up to time *t*^′^−*t* required for reaching *ϕ*. The first part can be compared to **F**_[0,*t*]_*ψ* and the second part corresponds to $\phantom {\dot {i}\!}\psi \mathbf {U}_{[0,t'-t]} \phi $:

$${{ \begin{aligned} {} Prob(M, s, \psi \mathbf{U}_{[t,t']} \phi) =\! \Sigma_{\substack{s \in Sat(\neg \psi)\\ s' \in Sat(\psi)}} \mathbf{P}_{t}(s,s') \cdot Prob(M,s',\psi \mathbf{U}_{[0,t'-t]} \phi) \end{aligned}}} $$

The last case *I*= [*t*,*∞*] is similar to the previous *I*= [*t,t*^′^] but changing $\psi \mathbf {U}_{[0,t'-t]} \phi \phantom {\dot {i}\!}$ by *ψ***U***ϕ* due to the infinity. As the until operator is unbounded, it is evaluated in the embedded DTMC:

$${{ \begin{aligned} {} Prob(M, s, \psi \mathbf{U}_{[t,\infty]} \phi) =\! \Sigma_{\substack{s \in Sat(\neg \psi) \\ s' \in Sat(\psi)}} \mathbf{P}_{t}(s,s') \cdot Prob(emb(M),s',\psi \mathbf{U} \phi) \end{aligned}}} $$

The temporal complexity of verifying a CSL^TA^ formula *ϕ* against a CTMC is linear in the number of logical connectives and temporal operators of the formula (|*ϕ*|) and polynomial in the size of *S*. More generally, the complexity is:

$$\Theta (poly(size(S))*q * t_{max}*|\phi|) $$

where *t*_*max*_ is the maximal bound of a path subformula *ψ*_1_**U**_*I*_*ψ*_2_ of *ϕ*, with *t*_*max*_=1 if it doesn’t contain any **U** subformula. The parameter *q* is equal to *q*=*max*_*s*∈*S*_|**Q**(*s,s*)|.

#### Model checking tools

A model checking tool or verifier requires two input files for the verification process: a first file with the description of the model, and a second file with the specification of the properties. Traditionally, it returns if the specification is satisfied in the model or not, and a counterexample that falsifies it if required. In probabilistic model checking, extra information shall be provided such as the minimum probability for which the property succeed.

The modularity and independence of model and specifications allow the evaluation of several properties over the same model and, vice versa, the test of the same phylogenetic requirements over different computational models. The encapsulation of the underlying technological implementation details (e.g., multi-threading, data bases, etc.) is one of the most important features of this kind of tools. The main advantages of the model checking framework are the abstract description of model and properties using logics and mathematical formalisms, and the portability of these files between computers that have installed the same model checking tool.

There exists a considerable diversity of verifiers with different performances, qualities and designs [34]. Among all the model checking tools developed for probabilistic systems, PRISM [35], MRMC [36] and CADP [37] are the most important ones.

In our case, we have selected PRISM for the experimentation for several reasons. PRISM is a generic state-based model checking tool capable of handling probabilistic and timed specifications over Markov chains. PRISM offers Java portability, a powerful syntax for handling time and probabilities in models and specifications, and a good scientific community support. Besides, it is open source, which allows the modification and optimization of its code. The real performance depends on the particular structure of the model and specifications. This fact is shown in the example of case of study.

### Case of study: maximum likelihood of a phylogenetic tree

Formal methods help for describing mutation models and evaluating properties over phylogenetic trees. Among all the potential applications, we focus on the problem of analyzing the statistical probability of a phylogeny. Maximum likelihood is one of the most common methods for phylogenetic inference. Given a mutation model, it scores the quality of the topology for a inferred tree. However, the incorporation of new models into current software tools requires an expertise in programming languages and a deep knowledge of the source code. This fact limits the extension, upgrade, debugging and maintenance of a software tool with new models defined by the biologists.

To this end, we show how to define a tree and a mutation model using the description language of CSL^TA^ and CTMCs. A phylogenetic tree is formulated as a succession of nucleotide mutations that are checked over a mutation model. The verification process returns the likelihood of obtaining the tree from a particular model. We have analyzed several phylogenies using the PRISM model checking tool. In addition, we have adapted the software for optimizing the computation of maximum likelihoods.

#### Translation to PRISM

Our framework plays the role of *worker* for computing the maximum likelihood during the evaluation of the tree topology in current inference algorithms and tools ([38], Fig. 2). The model checking tool returns a confidence value between [0,1], which represents the likelihood of getting a peculiar arrangement of nucleotides in the states of the tree with respect to a model of mutations. The score provided by the result of the verification process is useful for guiding the iterative refinement of the phylogenetic tree. Our tool requires an external tree generator.

**Fig. 6:**
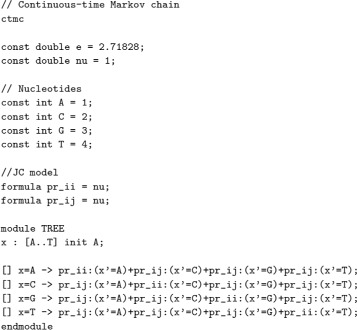
Description of the Jukes-Cantor model in PRISM syntax

The model checking tool requires as input a) a description of the DNA mutation model, and b) the specification of the tree in terms of likelihood equations. Figure 6 corresponds to the file describing the mutation model over which the phylogenetic tree will be evaluated. By changing the relations and the ratio of transitions, it is possible to switch from one mutation model to another. The logic selected for specifying properties and the abstract language for describing Markov chains improve the legibility of the system and make the maintenance easier. PRISM provides an user-friendly textual language for writing the model and equations.

The computation of the probability is model-dependent. Some of the historical mutation models are Jukes-Cator (JC), Kimura, Felsenstein or the Generalized time-reversible (GTR) [39]. For simplicity in this example, we consider the JC model. The substitution rate *nu* equals to 1. It expresses the number of ticks needed for the activation of the transition rather than an explicit probability.

**Fig. 7:**
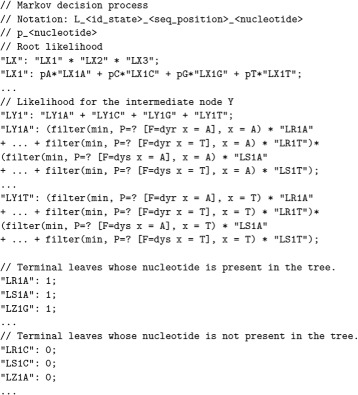
Representation in PRISM syntax of the MLE equations for Fig. 4

The translation of the mathematical equations to the syntax of the stochastic logic supported by PRISM is exemplified in Fig. 7. It corresponds to the MLE equations defining the tree of Fig. 4. The conversion is dealt by a BioPerl script [40]. The unfolding of these equations depends on the structure of the phylogenetic tree and the genome placed in its tips. In an indirect way, the equations *specify* the structure of phylogenetic tree with respect to a DNA mutation model: they depict the trace of mutations from the root to the leaves.

Besides asking for a particular bound, PRISM includes iterative methods for calculating maximal (minimal) probabilities of a path of the tree. For example, the operator P=?[F<=5 x1 = A] returns the probability of reaching a nucleotide A at position x1 of the alignment within 5 time steps in the future. It is equivalent to $\mathbb {P}_{=?}[\mathbf {F}_{<=5} seq[1] = A]$ in CSL^TA^ syntax, but ? is the threshold calculated by the model checking tool instead of an explicit probability.

PRISM allows the specification of the initial state. For example, filter(min, P=? [F<=5 x1 = A], x1 = C) returns the minimum probability taking the sequence x1 = C as the initial state. It ensures that there exists a path starting in x1 = C that eventually reaches x1 = A. The properties are annotated with names for defining the partial likelihoods $L^{l}_{X,i}$. In case of phylogenetic trees with estimated nucleotides in the ancestors (such as in the case of maximum parsimony), the intermediate nodes are initialized with constant values $L^{l}_{X,i}=1$ or $L^{l}_{X,i}=0$ for reducing the initial calculations. The header of the file describes the notation of the variable names. The constant dxy is the distance between states X and Y.

However, the current release of PRISM does not store the partial results $L^{l}_{X,i}$ in local memory, which implies a reevaluation of $L^{l}_{X,i}$ every time it is accessed. The inability for caching these values damages the potential optimization caused by the factorization of the probabilities *P*_*ij*_(·) in Eq. 3. This peculiarity, together with the inherent delay introduced by the Java virtual machine, penalizes the performance in comparison to other specific systems for computing maximum likelihoods [22, 23]. In order to solve this problem, we have modified PRISM for caching the partial results $L^{l}_{X,i}$. Applying this simple change, the model checker slightly improves its performance.

#### Experimentation

**Fig. 8:**
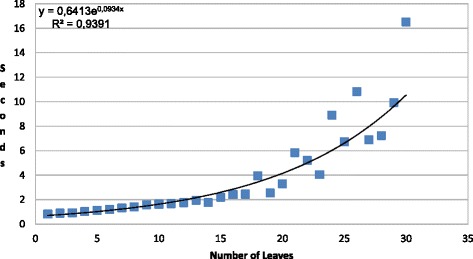
Time required in PRISMopt for the evaluation of the maximum likelihood equations

Figure 8 presents the experimental results with the adapted version of PRISM. The data set used for this experimentation is synthetic. Using this data set, we try to cover the spectrum of small phylogenies and analyze the cost of the evaluation of the MLE equations over there. We have created random phylogenetic trees of up to 30 tips using a Yule backward model [20]. For each tree size (number of tips), we have generated ten random trees and calculated the harmonic mean time. The genomes have a single base with an homogeneous distribution of nucleotides. Although subject to a perfect parallelism, the temporal cost for larger strings can be estimated linearly by multiplying the time by the number of bases.

All tests have been run on a Intel Core 2 Duo E6750 @ 2.66 GHz with 8 GB RAM and Linux. Memory is not a limiting factor because the experiments have been successfully executed with only 4GB assigned to the Java virtual machine. The factorization of the equations and the initialization cost in PRISM are delays that must be considered for the judgment of the results.

The exponential trend observed in the graphic is explained by the exploration method selected by PRISM for calculating the probability function *P*_*ij*_(*d*). In this way, PRISM unfolds the DNA mutation model and generates all the possible traces between the bases *i* and *j*, applying implicitly an exhaustive searching method. PRISM looks for all the paths of *n* nodes with distance *Σ*_*k*=1…*n*_*d*_*k*_=*d* that maximize *P*_*ij*_(*d*), being *i* (*j*) the initial (final) state and *d*_*k*_ the distance between each pair of intermediate states. The combinatorial exploration of the paths satisfying the previous restrictions of length and probability leads to the exponential cost for calculating *P*_*ij*_(*d*).

**Fig. 9:**
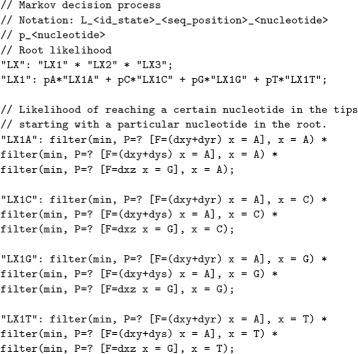
Rewriting of the MLE equations for Fig. 4

We propose an alternative configuration of the equations in order to take advantage of the peculiar procedure in PRISM for managing probabilities. Figure 9 shows this rewriting. Now, exclusively the phylogenetic leaves are assigned with a DNA value because they are the only values whose nucleic base we know with certitude. The internal nodes are left undetermined in the specification. The addition of the distances dxy+dyr marks the length of the path between the root and the leaf R. The estimation resulting from the evaluation of the formulas in Fig. 9 places an upper bound to the real value of likelihood. Each filter extracts independent paths with maximum probability, presumably giving rise to a set of disjoint routes whose only common ancestor is the root. Although probably depicting a degenerated phylogenetic tree, it results in a suitable heuristic for pointing out the maximum likelihood.

**Fig. 10:**
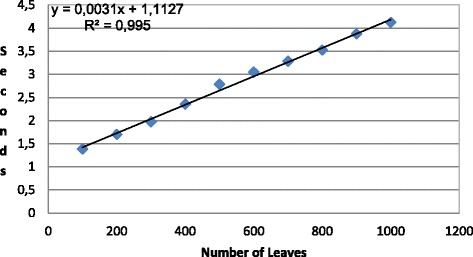
Time required in PRISM for the computation of the upper bound of the maximum likelihood value

Figure 10 presents the performance results of the experiments. We continue using the same methodology introduced in previous trials. We have created random phylogenetic trees of up to 1000 tips using a Yule backward model [20]. For each tree size (number of tips), we have generated ten random trees and calculated the harmonic mean time. The genomes have a single base with an homogeneous distribution of nucleotides. The main consequence of the new configuration in the equations is a linear trend in the temporal cost. It grants the evaluation of bigger phylogenies and larger sequences than before.

This framework becomes competitive for obtaining an upper-bound approximation for MLE efficiently. Future optimizations should focus on the implementation of more efficient libraries and dynamic programming techniques that solve, or at least ease, the aforementioned limitations and bottlenecks in PRISM.

## Conclusions

Model checking is a generic framework that has been applied for qualitative analysis over phylogenies. The main advantages of this approach are the legibility and soundness of formal methods based on temporal logics, the independence of the model from the specifications and the availability of powerful verifiers that automatically analyze the specifications and find counterexamples. Besides, the encapsulation of the implementation in a model checking tool hides and simplifies the access to the underlying technology.

Nevertheless, some phylogenetic properties are beyond the expressiveness of first-order temporal logics used in that environment. In this paper, we have completed the previous framework including explicit time and probabilistic information. To this end, we have applied stochastic extensions of the model checking framework for querying quantitative properties over phylogenetic trees and mutation models. The inclusion of explicit time and probabilities matches naturally with the speciation models. In this sense, we have introduced a stochastic logic, data structure and methods adapted for manipulating and computing probabilities over continuous-time systems. Several quantitative properties have been specified.

In order to prove the feasibility of our approach, we focused on the problem of analyzing the statistical probability of a phylogeny. We have studied the likelihood of obtaining a phylogenetic tree through the evaluation of maximum likelihood estimations over DNA mutation models. A phylogenetic tree is formulated as a set of paths using temporal logics (i.e., a path is understood as a succession of nucleotide mutations that are checked over a mutation model). The verification process returns the likelihood of obtaining that tree from a mutation model.

We have shown how to translate the model and specifications to the particular notation of this framework. We have analyzed several synthetic (random) phylogenies using the PRISM model checking tool. A linear-time heuristic has been proposed for the calculation of an upper bound of the likelihood score. We have customized the tool in order to slightly improve the verification costs.

This work opens the door for the review of bigger systems with quantitative properties similar to those defined in this paper. The modularity of our framework allows the evaluation of hypotheses and the comparison of results for a set of phylogenetic trees by only changing the tree file (the specification of the property remains constant). Finally, the search for the valuations that verify a certain specification leads to an intensive exploration of the formula space or the solution of linear systems. The introduction of parametric model checking for the automatic discovery and mining of phylogenetic information outlines our future work.

## Abbreviations

AP, atomic proposition; BM, back mutation; CADP, construction and analysis of distributed processes; CSL^TA^, continuous stochastic logic with timed automata; CTL, computational tree logic; CTMC, continuous-time Markov chain; DTMC, discrete-time Markov chain; GTR, generalized time-reversible; JC, Jukes-Cator; MLE, maximum likelihood estimation; MRMC, Markov reward model checker; PCTL, probabilistic CTL; PTS, probabilistic transition system
